# The intrinsically disordered tails of PTEN and PTEN-L have distinct roles in regulating substrate specificity and membrane activity

**DOI:** 10.1042/BJ20150931

**Published:** 2016-01-05

**Authors:** Glenn R. Masson, Olga Perisic, John E. Burke, Roger L. Williams

**Affiliations:** * Medical Research Council, Laboratory of Molecular Biology, Cambridge CB2 0QH, U.K.

**Keywords:** hydrogen/deuterium exchange mass spectrometry (HDX–MS), interfacial catalysis, intrinsically disordered protein regions, phosphatase and tensin homologue deleted on chromosome 10 (PTEN), (PTEN-L)

## Abstract

Phosphatase and tensin homologue deleted on chromosome 10 (PTEN) is a lipid and protein phosphatase, and both activities are necessary for its role as a tumour suppressor. PTEN activity is controlled by phosphorylation of its intrinsically disordered C-terminal tail. A recently discovered variant of PTEN, PTEN-long (PTEN-L), has a 173-residue N-terminal extension that causes PTEN-L to exhibit unique behaviour, such as movement from one cell to another. Using hydrogen/deuterium exchange mass spectrometry (HDX–MS) and biophysical assays, we show that both the N-terminal extension of PTEN-L and C-terminal tail of PTEN affect the phosphatase activity using unique mechanisms. Phosphorylation of six residues in the C-terminal tail of PTEN results in auto-inhibitory interactions with the phosphatase and C2 domains, effectively blocking both the active site and the membrane-binding interface of PTEN. Partially dephosphorylating PTEN on pThr^366^/pSer^370^ results in sufficient exposure of the active site to allow a selective activation for soluble substrates. Using HDX–MS, we identified a membrane-binding element in the N-terminal extension of PTEN-L, termed the membrane-binding helix (MBH). The MBH radically alters the membrane binding mechanism of PTEN-L compared with PTEN, switching PTEN-L to a ‘scooting’ mode of catalysis from the ‘hopping’ mode that is characteristic of PTEN.

## Abstract (precis)

The unstructured regions found at the C-terminus of the tumour suppressor PTEN and the N-terminus PTEN-L can switch the enzymes' substrate specificity from soluble to membrane-embedded, and can also dramatically alter the enzymes' affinity for membranes.

## INTRODUCTION

PTEN (phosphatase and tensin homologue deleted on chromosome 10) is a tumour suppressor, with inactivating mutations that are among the most common in solid tumours [1–3]. Although initially identified as a protein tyrosine phosphatase (PTP) [4], the preferred substrate of PTEN is the lipid second messenger phosphatidylinositol 3,4,5-trisphosphate (PIP_3_) [5]. PTEN activity directly opposes the activity of the phosphoinositide 3-kinases (PI3Ks), preventing activation of the master protein kinase Akt and consequently inhibiting cell growth and proliferation [6,7]. PTEN can also antagonize the PI3K pathway as a protein phosphatase through dephosphorylation of insulin receptor substrate 1 (IRS1) [1–3,8]. Homozygous PTEN knockouts are lethal in mice, and heterozygous mutations result in a predisposition to tumour formation [4,9,10]. Characterization of PTEN mutations in tumours has led to a continuum model for tumour suppression in which loss of a single PTEN allele is sufficient to drive tumorigenesis [5,11]. More recently, catalytically compromised PTEN mutants have been proposed to act in a dominant-negative mechanism due to dimerization [6,7,12], causing dramatic decreases in PTEN activity even when a single allele is mutated.

**Figure 1:**
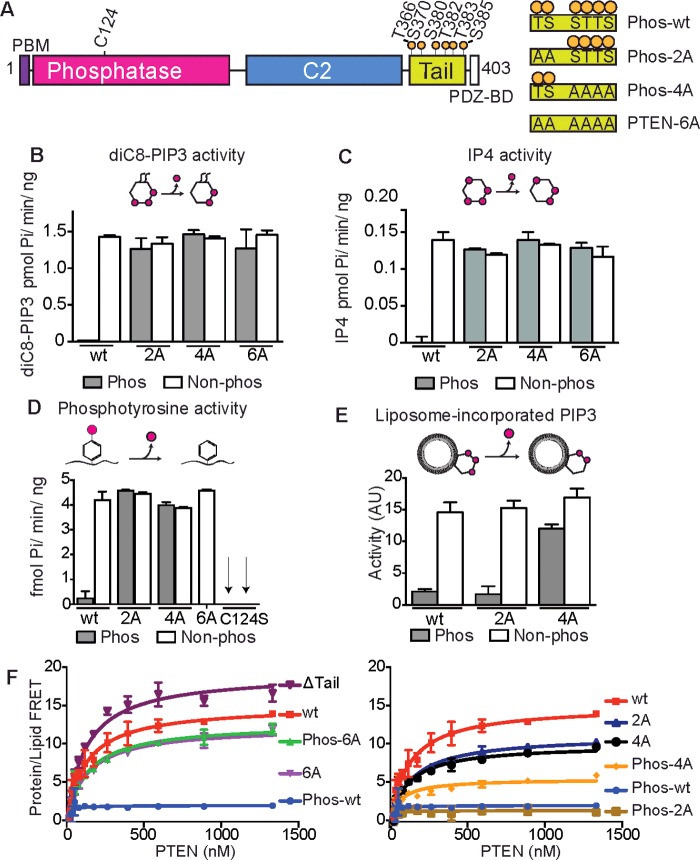
Phosphorylation of PTEN C-terminal tail regulates its activity and substrate specificity (**A**) Schematic representation of PTEN, highlighting various phosphorylation sites. The PBM (residues 1–7) is followed by a phosphatase domain (14–185), the C2 domain (190–351), the 50-amino-acid tail (353–400) containing multiple phosphorylation sites and the PDZ-binding motif (401–403). Differentially phosphorylated constructs of PTEN were produced, referred to as Phos-wtPTEN, Phos-PTEN-4A and Phos-PTEN-2A. (**B**) Phosphatase activity of PTEN against soluble diC_8_-PIP_3_ substrates. All activity assays and lipid-binding assays have error bars showing S.D. from the mean, derived from experiments carried out in triplicate. Some error bars are smaller than the marker. All experiments were conducted at least three times. (**C**) Phosphatase activity against IP_4_, a soluble head group substrate. (**D**) Phosphatase activity against a phosphotyrosine-containing polypeptide. (**E**) Phosphatase activity against lipid vesicles containing a long-chain PIP_3_ substrate. PIP_2_-containing vesicles (5%) were incubated with PI3K to generate PIP_3_, which was subsequently dephosphorylated with various concentrations of PTEN. (**F**) Protein/lipid FRET curves of variously phosphorylated PTEN constructs binding to 5% PIP_2_-containing membrane-mimicking vesicles.

PTEN is composed of three domains flanked by two motifs (Figure 1A): an N-terminal phosphatidylinositol 4,5-bisphosphate (PIP_2_)-binding motif (PBM, residues 1–7), a dual-specificity phosphatase domain, a C2 domain, a 50-amino-acid unstructured C-terminal tail and a C-terminal PDZ-binding motif [13,14]. The phosphatase domain has an active site that is formed by the conjunction of three loops: the P loop, the WPD loop and the TI loop. The WPD loop (residues 88–98) is a flexible loop that carries Asp^92^, a residue critical for substrate catalysis [13,15–17]. The TI loop (residues 160–171) forms a uniquely deep pocket to accommodate the bulky PIP_3_ substrate [13,16]. The highly conserved P loop (residues 121–131) contains the HCXXGXXR phosphatase signature motif, including the catalytically crucial cysteine, Cys^124^, which forms a cysteinyl phosphoenzyme intermediate that is subsequently hydrolysed [18].

PTEN activity is controlled epigenetically [19], post-transcriptionally and post-translationally [20]. The 47-kDa PTEN protein is subject to phosphorylation [21,22], acetylation [23], oxidation [24], ubiquitination [25] and SUMOylation [26]. Furthermore, recent discoveries have shown that PTEN can be transported from one cell to another, via ubiquitin-mediated exosomal sorting [27] and through secretion of PTEN-long (PTEN-L) [28,29].

The N-terminal extension of PTEN-L (a longer variant of PTEN which results from an alternative translation initiation site) encompasses a poly-alanine secretion signal and a poly-arginine re-entry sequence that enable the protein to be secreted and enter neighbouring cells respectively [29]. PTEN-L also localizes to the mitochondria, where it regulates cytochrome *c* oxidase function [30]. The N-terminal extension is predicted to be highly disordered and the target of numerous post-translational modifications [31]. PTEN-L was suggested to be in a constitutively active state with regard to PIP_2_-mediated activation of PTEN, possibly due to an altered structure surrounding the PBM [32].

PTEN activity is closely regulated by phosphorylation at multiple sites by serine/threonine kinases. Six inhibitory phosphorylation sites are located within the PTEN tail on residues Thr^366^, Ser^370^, Ser^380^, Thr^382^, Thr^383^ and Ser^385^ (Figure 1A). These sites are targeted by the CK2 and GSK3β [22,33–35], preventing proteasomal degradation of PTEN [36]. Two models of auto-inhibition have been proposed. In one model, the phosphorylated tail interacts with the calcium-binding region (CBR)3 loop of the C2 domain, either electrostatically [35] or non-electrostatically [37], preventing membrane binding and PIP_3_ dephosphorylation. The second model argues that the phosphorylated tail interacts with the phosphatase domain and the PIP_2_-binding domain (PBD) [22]. Auto-dephosphorylation of pThr^366^ and pSer^370^ can selectively remove these phosphates [38].

In the present study, we determine how phosphorylation of PTEN and the addition of the N-terminal extension of PTEN-L affect enzymatic properties such as substrate specificity and interfacial catalysis. Furthermore, we use HDX–MS to provide structural insights into how the phosphorylated C-terminal tail of PTEN and a putative α-helix in the N-terminal extension of PTEN-L regulate these catalytic properties.

## MATERIALS AND METHODS

### Protein expression and purification

For expression of recombinant PTEN proteins, 1–8 litres of *Spodoptera frugiperda* Sf9 cells at a density of 1×10^6^ cells/ml were infected with an optimized ratio of virus. Expression of PTEN in *Escherichia coli* was found to produce unsatisfactory material that showed pronounced aggregation. All PTEN constructs contain an N-terminal His_6_-tag followed by a tobacco etch virus (TEV) cleavage site. After 48 h of infection at 27°C, the cells were harvested and washed with ice-cold PBS. The pellets were lysed by sonicating for 5 min in Buffer A [20 mM Tris/HCl, pH 8.0, 300 mM NaCl, 10 mM imidazole, 5% glycerol, 2 mM 2-mercaptoethanol and 0.5% Triton X-100, with one Complete EDTA-free protease inhibitor tablet added (Roche) per 50 ml of buffer]. Lysate was centrifuged for 45 min at 140000 ***g***. The supernatant was then passed through a 0.45 μm filter (Sartorius Biotech) and then loaded on to a 5 ml of HisTrap FF column (GE Healthcare). The column was washed with up to 30 mM imidazole before being eluted with a 0–100% gradient of Buffer B (20 mM Tris/HCl, pH 8.0, 100 mM NaCl, 5% glycerol, 200 mM imidazole and 2 mM 2-mercaptoethanol). The pooled fractions containing the PTEN were then placed along with an aliquot of His-tagged TEV protease into a 10 000 Da molecular-mass cut-off Snakeskin Dialysis membrane and dialysed in 4 litres of Buffer C (20 mM Tris/HCl, pH 8.0, 200 mM NaCl, 5% glycerol, 2 mM tris-(2-carboxyethyl(phosphine (TCEP)) at 4°C for 12 h. The ratio of PTEN/TEV in the dialysis was 20:1 (w/w). To remove the His_6_–TEV protease, the dialysed solution was subsequently passed back over a 5 ml of HisTrap FF column. Flow-through from the HisTrap column was then diluted 1:1 with Buffer D (20 mM Tris/HCl, pH 8.0, 10% glycerol and 1 mM DTT), before being loaded on to a 5 ml HiTrap Q column (GE Healthcare) pre-equilibrated in Buffer E (20 mM Tris/HCl, pH 8.0, 50 mM NaCl, 10% glycerol and 1 mM DTT). Protein was eluted with a gradient of 0–100% Buffer F (20 mM Tris/HCl, pH 8.0, 1 M NaCl, 10% glycerol and 1 mM DTT). Pooled fractions were concentrated with an Amicon 10 000 Da molecular-mass cut-off centrifugal filter (Millipore) and injected on to a Superdex 75 16/60 gel-filtration column equilibrated in Buffer G (20 mM HEPES, pH 7.4, 200 mM NaCl and 2 mM TCEP). Fractions were collected and concentrated to at least 1.5 mg/ml using an Amicon 10 000 Da molecular-mass cut-off centrifugal filter (Millipore) then divided into aliquots, frozen in liquid nitrogen and stored at–80°C.

PTEN-L was expressed with the six C-terminal phosphorylation sites mutated to alanine (PTEN-L-6A; T539A-L, S543A-L, S553A-L, T555A-L, T556A-L and S558A-L), as all dephosphorylation conditions tested were found to cause the protein to precipitate. PTEN-L-6A was expressed with an N-terminal TEV-cleavable Protein A tag in Sf9 cells at 27°C for 41 h, before being harvested as above. The pellets were lysed by sonicating 5 min in Buffer A-L [20 mM Tris/HCl, pH 8.0, 300 mM NaCl, 5% glycerol, 2 mM 2-mercaptoethanol, 0.1% Triton X-100 and 0.5% CHAPS, with one Complete EDTA-free protease inhibitor tablet added (Roche) per 50 ml of buffer]. Lysate was centrifuged for 45 min at 140000 ***g***. The supernatant was then passed through a 0.45 μm filter (Sartorius Biotech). IgG beads (GE Healthcare, catalogue number 17096902) were added to the supernatant and incubated for 2 h at 4°C. The beads were then washed with 150 ml of wash buffer (50 mM Tris/HCl, pH 8.0, 300 mM NaCl, 2 mM TCEP, 0.1% Triton X-100 and 0.5% CHAPS), followed by 150 ml of TEV buffer (50 mM Tris/HCl, pH 8.0, 300 mM NaCl and 2 mM TCEP). PTEN-L-6A was then removed from the beads by incubation with TEV protease overnight, followed by elution using three 15-ml washes with TEV buffer. PTEN-L-6A was then loaded on to a HiTrap SP HP column equilibrated in Buffer SA (20 mM HEPES, pH 7.5, 50 mM NaCl and 1 mM DTT) and eluted using a gradient of 0–100% Buffer SB (20 mM HEPES, pH 7.5, 1 M NaCl and 1 mM DTT).

### Protein phosphorylation

Protein was incubated at room temperature for 24 h with glycogen synthase kinase 3β (GSK3β) (New England Biolabs) and protein kinase CK2 as per the manufacturer's instructions (approximately 10000 units of each kinase per 1000 μl of 40 μM PTEN). The reaction mixture was injected on to a Superdex 75 10/300 gel-filtration column (or a Superdex 75 16/600 for larger volumes) pre-equilibrated in Buffer G. Fractions containing protein were concentrated and frozen in liquid nitrogen for subsequent analysis. Phosphorylation status was checked with both intact and peptide fragmentation MS.

### Protein dephosphorylation

Protein was incubated at 30°C for 90 min with λ-phosphatase as per the manufacturer's instructions (approximately 50000 units per reaction). The reaction mixture was then injected on to a Superdex 75 10/300 gel filtration column pre-equilibrated in Buffer G. Fractions containing protein were concentrated and frozen in liquid nitrogen for subsequent analysis. Phosphorylation status was checked with both intact and peptide fragmentation MS.

### Intact MS

PTEN samples (60 μl at 0.4 μM) were injected on to an ultra-performance liquid chromatography (UPLC) system and passed over a C_4_ Van-Guard pre-column (Waters) in 0.1% formic acid for 3 min to remove excess salt before elution using a 3λ-p–100% B gradient of Buffer A (0.1% formic acid) and Buffer B (100% acetonitrile) over 13 min. This eluent was injected on to a Xevo QTOF (Waters) acquiring a mass range from 350 to 1500 *m*/*z*, with an ESI source operated at a temperature of 225°C and a spray voltage of 2.5 kV. Data were deconvoluted using MaxEnt Software (MicroMass).

### Soluble lipid phosphatase activity assay

Recombinant PTEN was incubated with 50 μM dioctanoyl-PIP_3_ (diC_8_-PIP_3_) (Echelon) in 50 mM Tris/HCl, pH 8.0, and 2 mM DTT for 10 min at 37°C in a final volume of 25 μl. The reaction was stopped by the addition of 100 μl of Malachite Green Reagent (Millipore). The reaction was then incubated at room temperature, with agitation at 400 rev./min for 15 min to allow colour to develop. The absorbance at 620 nm was measured and the amount of phosphate released was determined by using a KH_2_PO_4_ (Millipore) standard curve. The specific activity of the PTEN was obtained by dividing the amount of phosphate released by the concentration of the enzyme.

### Lipid vesicle preparation

Lipid components were mixed together while in organic solvent (chloroform or chloroform/methanol). The solvent was then evaporated under a stream of nitrogen. Vesicles were composed of 5% brain PIP_2_, 20% brain phosphatidylserine (PS), 45% brain phosphatidylethanolamine (PE), 15% brain phosphatidylcholine (PC), 10% cholesterol and 5% sphingomyelin (w/v; Avanti Polar Lipids). The lipid film was allowed to dry for 1 h under vacuum and then resuspended in 20 mM HEPES, pH 7.4, 100 mM KCl and 1 mM EGTA. The lipids were bath sonicated for 10 min then subjected to ten freeze–thaw cycles between liquid nitrogen and a 43°C water bath. The liposomes were finally extruded 11 times through a 100-nm filter. Vesicles were frozen in liquid nitrogen and stored at–80°C, before being defrosted at room temperature before use.

### Protein/lipid FRET assays

Vesicles were prepared exactly as for the lipid kinase assays, except that 10% dansyl-PS was substituted for 10% of the brain phosphatidylserine. Vesicles were diluted with 20 mM HEPES, pH 7.4, 100 mM KCl and 1 mM EGTA to give a final concentration of 50 μg/ml. PTEN was thawed and centrifuged at 13000 rev./min at 4°C to remove any precipitate. PTEN was diluted in 20 mM HEPES, 200 mM NaCl and 2 mM TCEP to a final concentration of 4 μM. Protein was serially diluted in a 2:3 ratio in the above buffer. The lipid solution (5 μl) was then mixed with 5 μl of the protein solution at various concentrations. The protein/lipid mixture was allowed to equilibrate for 10 min, while being agitated at 450 rev./min at room temperature. Reactions were measured with a PHERAStar HTS microplate reader (BMG Labtech) using a 280 nm excitation filter with 350- and 520-nm emission filters to measure tryptophan and dansyl-PS FRET emissions respectively. The FRET signal is *I–I*_o_, where *I* is the intensity at 520 nm and where *I*_o_ is the intensity of the solution containing only lipid (i.e. without protein). Binding curves were fitted with a one site-specific-binding model (GraphPad Prism version 5.00).

### PIP_3_ incorporated lipid vesicle phosphatase assay

PIP_2_-containing vesicles with the composition described above were resuspended at a concentration of 1 mg/ml and phosphorylated by incubation for 2 h with PI3K, purified as detailed previously [42]. The reaction was carried out in 20 mM Tris/HCl, pH 7.4, 50 mM NaCl, 50 mM KCl, 1 mM EGTA, 3 mM MgCl_2_ and 0.1 mM ATP, before being quenched with 10 mM EDTA. The resulting PIP_3_-containing vesicles were then incubated with PTEN for 3 min at 25°C and quenched with 22 mM H_2_O_2_ (Sigma). The quenched reaction was agitated for 10 min at 500 rev./min at 25°C, before being spotted on to a nitrocellulose membrane. After the droplet had dried, the membrane was washed four times with TBS/Tween 20 solution (TBST) (50 mM Tris/HCl, pH 7.4, 150 mM NaCl and 0.1% Tween 20) over the course of 1 h, before being blocked with TBST+2 mg/ml BSA for 1 h at 4°C. The membrane was then incubated with 0.5 μg/ml Alexa Fluor 488-labelled GRP1-PH domain in TBST+2 mg/ml BSA overnight at 4°C as described previously [58]. The membrane was washed six times in TBST and dried at 25°C before being imaged using a Typhoon imager (GE Healthcare). The intensities were quantified using ImageQuant (GE Healthcare).

### Surface dilution assay

The assay was conducted as described above, but with two lipid stock solutions, a 1 mg/ml 5% PIP_2_ plasma-membrane-mimicking composition and a 2 mg/ml 2.5% PIP_2_ plasma-membrane-mimicking composition. PIP_3_ was generated as above. Reactions were conducted with 10 nM PTEN over a 40-min period. PIP_2_ levels were determined using a GRP1-PH domain as described above.

### Hydrogen/deuterium exchange (HDX) MS

HDX reactions were conducted with 10 μl of protein in dilution buffer (20 mM HEPES, pH 7.5, 150 mM NaCl and 2 mM TCEP) and initiated by the addition of 40 μl of D_2_O buffer solution (10 mM HEPES, pH 7.5, 50 mM NaCl, 2 mM TCEP and 92% D_2_O), to give a final concentration of 74% D_2_O. Final protein concentrations were 1 μM. For HDX–MS membrane interaction experiments, lipid vesicles were mixed with the D_2_O buffer. Four time points of exchange (3, 30, 300 and 3000 s) were terminated by the addition of a quench buffer (final concentration 0.6 M guanidinium chloride and 0.8% formic acid). Samples were rapidly frozen in liquid nitrogen and stored at–80°C until MS analysis.

### Measurement of deuterium incorporation

Protein samples were rapidly thawed and injected on to a UPLC system immersed in ice as previously described [42]. The protein was run over an immobilized pepsin column (Applied Biosystems; Poroszyme, catalogue number 2-3131-00) at 130 μl/min for 3 min and the peptides were collected on to a Van Guard pre-column trap (Waters). The trap was subsequently eluted in line with an Acquity 1.7 μm particle, 100 mm×1 mm C_18_ UPLC column (Waters), using a gradient of 5–36% B (buffer A 0.1% formic acid, buffer B 100% acetonitrile) over 20 min. Eluent from the column was injected on to a Xevo QTOF (Waters) acquiring over a mass range from 350 to 1500 *m*/*z* for 30 min, using an ESI source operated at a temperature of 225°C and a spray voltage of 2.5 kV.

### Protein digestion and peptide identification

Peptide identification was done by running MS/MS experiments using a 5–36% B gradient over 120 min with a Xevo QTOF (Waters). This was supplemented with a 20-min gradient separation to identify and correct the retention time for all samples. The MS tolerance was set to 3 ppm with an MS/MS tolerance at 0.1 Da. The resulting MS/MS datasets were analysed with the Mascot search within Proteome Discoverer (Thermo Scientific). All peptides with a Mascot score >20 were analysed using HD-Examiner Software (Sierra Analytics). The full list of peptides was then manually validated by searching a non-deuterated protein sample's MS scan to test for the correct *m*/*z* state and check for the presence of overlapping peptides. Ambiguously identified peptides were excluded from all subsequent analysis. The first round of analysis and identification were performed automatically by the HD-Examiner software, but all peptides (deuterated and non-deuterated) were manually verified at every state and time point for the correct charge state, *m*/*z* range, presence of overlapping peptides and any deviation from the expected retention time. All HDX experiments were carried out in triplicate.

### Mass analysis of peptide centroids

All results are presented as relative levels of deuterium incorporation and no correction for back exchange is applied, because no fully deuterated protein sample could be obtained. All percentage differences quoted in the Results section are the maximal changes in HDX seen at any time point of the analysis, unless explicitly stated otherwise.

### Size-exclusion chromatography–multi-angle light scattering (SEC–MALS)

PTEN solution (100 μl of 20–50 μM protein) was injected on to a Superdex 200 GL 10/300 gel-filtration column (GE Healthcare) equilibrated in 20 mM HEPES, pH 7.4, 200 mM NaCl and 2 mM TCEP. Samples were run at 0.5 ml/min and eluted into a Dawn Heleos (Wyatt) (for light-scattering measurements) and Optilab rEX (Wyatt; for refractive index measurements). The system was calibrated using a BSA standard prior to samples being run.

## RESULTS

### Phosphorylation of the C-terminal tail differentially inhibits PTEN towards distinct substrates

PTEN was expressed in *Spodoptera frugiperda* Sf9 cells and purified to determine how the protein phosphatase and lipid phosphatase activities of PTEN were regulated by post-translational modifications of the C-terminal tail. To produce homogeneously and selectively phosphorylated PTEN, several constructs were produced. First, a construct containing six mutations, T366A, S370A, S380A, T382A, T383A and S385A (PTEN-6A), was prepared to prevent background heterogeneous phosphorylation of the tail, as previously described [22,37,39] (Figure 1A). We also generated two constructs that preserved only a portion of the tail phosphorylation sites: PTEN-2A with mutations T366A and S370A (mimicking auto-dephosphorylated PTEN [38]) and PTEN-4A with mutations S380A, T382A, T383A and S385A. We also expressed both full-length PTEN as well as a truncated PTEN lacking the tail (PTEN-ΔTail). We used λ-phosphatase to dephosphorylate PTEN or CK2 and GSK3β kinases to phosphorylate it. PTEN constructs were further purified after λ-phosphatase or kinase treatment before biochemical and structural analysis. Proteins produced in this manner were homogeneously dephosphorylated or phosphorylated, as verified by LC–MS of intact proteins (Supplementary Figure S1). Using SEC–MALS, both the phosphorylated and the non-phosphorylated PTEN were shown to be monodisperse, with masses in agreement with what would be expected for monomers (Supplementary Figure S2).

To compare the activities and specificities of differentially phosphorylated PTEN constructs, phosphatase assays were conducted against four substrates: a phosphotyrosine-containing acidic polypeptide, the soluble lipid diC_8_-PIP_3_, inositol tetrakis phosphate (IP_4_) and liposome-incorporated PIP_3_ (Figures 1B–1E). The phosphatase activity was examined for each PTEN construct in either a phosphorylated or a dephosphorylated form. To verify that the phosphatase activity present in the dephosphorylated PTEN samples was solely due to PTEN, an inactive C124S-PTEN mutant, treated with λ-phosphatase, was used as a control to ensure that there was no measurable contaminating phosphatase activity.

We found that phosphorylation of PTEN caused a dramatic reduction in activity against all substrates. Intriguingly, when PTEN was phosphorylated on only four sites (pSer^380^/pThr^382^/pThr^383^/pSer^385^) by using the phosphorylated PTEN-2A mutant (Phos-PTEN-2A), activity against the phosphopeptide and soluble diC_8_-PIP_3_/IP_4_ substrates was restored (Figures 1B–1D), but PTEN remained inhibited against liposome-incorporated PIP_3_ substrate (Figure 1E; Supplementary Figure S3). This suggests that pThr^366^ and pSer^370^ may have a role in occluding the active site of PTEN, whereas pSer^380^, pThr^382^, pThr^383^ and pSer^385^ have a role in occluding the membrane-binding surface. Phos-PTEN-4A, phosphorylated on only two sites, pThr^366^ and pSer^370^, showed similar activities with all substrates to that of dephosphorylated PTEN-4A, with only a slight reduction in activity against liposome-incorporated PIP_3_ (Figures 1B–1E).

### Phosphorylation of the C-terminal tail inhibits membrane binding

To determine whether the substrate specificities of differentially phosphorylated PTENs are due to altered affinities for liposomes, we conducted protein/lipid FRET binding experiments [40] using liposomes with a composition mimicking plasma membranes [41] (Figure 1F). Dephosphorylated PTEN (subsequently referred to as wtPTEN) binds to these liposomes, but Phos-wtPTEN had no measurable membrane binding. PTEN-ΔTail maintained membrane affinity, and all dephosphorylated constructs showed an affinity similar to that of wtPTEN, suggesting that neither the tail nor the six threonine/serine residues have crucial roles in contacting the membrane. Phos-PTEN-4A (phosphorylated on Thr^366^ and Ser^370^) showed a slight decrease in membrane binding (Figure 1F) which may be due to electrostatic repulsion. Consequently, a slight reduction in liposome-incorporated PIP_3_ phosphatase activity was observed (Figure 1E). Phos-PTEN-2A (phosphorylated on Ser^380^, Thr^382^, Thr^383^ and Ser^385^) showed dramatically decreased membrane binding, exhibiting an affinity indiscernible from phosphorylated wtPTEN, accompanied with a great reduction in activity against liposome-incorporated PIP_3_.

### HDX–MS shows that the phosphorylated C-terminal tail forms intramolecular interactions with both the C2 and the phosphatase domains

**Figure 2:**
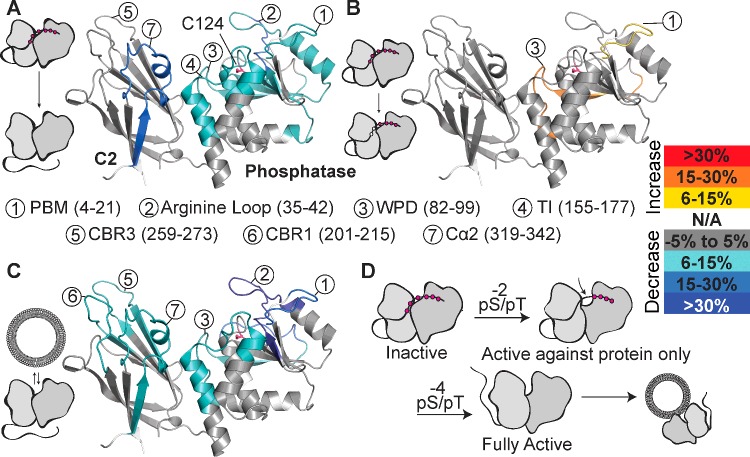
HDX-MS characterization of PTEN upon phosphorylation and membrane binding (**A**) Changes in HDX observed between wtPTEN phosphorylated by CK2/GSK3 and λ-phosphatase-treated PTEN. Only peptides with HDX changes greater than 0.7 Da and 6% are shown. All HDX experiments were carried out in triplicate. None of the loops missing from the crystal structure had any observed changes and are not shown. Areas with no peptide coverage are shown as white (N/A). (**B**) Two peptides, residues 4–21 (PBM) and residues 82–99 (WPD), showed an increased rate of HDX in S366A/T370A phosphorylated mutant compared with the wild-type phosphorylated protein, with the WPD loop reaching the same level of HDX as the dephosphorylated PTEN. (**C**) Changes in HDX observed with dephosphorylated wtPTEN in the presence of PIP_2_-containing vesicles. (**D**) Schematic representation of the phosphorylation-dependent mechanism of inactivation.

To understand the molecular details behind phosphorylation-mediated auto-inhibition, we carried out HDX–MS and compared the HDX levels of the dephosphorylated enzyme with those of the fully phosphorylated enzyme, as described previously [42]. Further details of the experiments can be found in the Supplementary Online Data (see Supplementary Figure S4 and the deuterium incorporation for all experiments are shown in Supplementary Figures S5–S8). The changes in exchange described in the text are the largest seen at any time point. Using HDX–MS it was possible to determine the location of conformational changes that occur upon tail phosphorylation (Figure 2A). Phosphorylation produced decreases in HDX (defined as changes greater than both 0.7 Da and 6% at any time point) in regions of phosphatase domain, as well as part of the C2 domain. In the phosphatase domain, five peptides (residues 4–21, 35–45, 82–99, 111–134 and 155–177) showed decreases in exchange. These peptides span the N-terminal PBM, a positively charged loop termed here the ‘arginine loop’ (the loop spanning pβ2-pα1, residues 35–49 [13]), the WPD loop, the P loop and the TI loop respectively. In the C2 domain, the Cα2 loop (residues 321–342) also shows a reduced HDX (peptide 319–341) upon phosphorylation. Overall, these decreases in HDX suggest that the fully phosphorylated C-terminal tail makes contact at the C2/phosphatase interface and more extensive contacts within the phosphatase domain, interacting extensively with all three loops of the active site and the PBM.

### Dephosphorylation of Thr^366^ and Ser^370^ exposes parts of the active site

We used HDX–MS to determine the molecular mechanism that allows selective activation of Phos-PTEN-2A (T366A, S370A) against soluble substrates but not lipid substrates residing within membranes by comparing the HDX rates of fully phosphorylated PTEN (Phos-wtPTEN) with Phos-PTEN-2A. We found that two peptides, residues 4–21 spanning the PBM, and residues 82–99, spanning the WPD loop, had increased rates of HDX (Figure 2B). The largest increase in exchange (>15%) occurs in the WPD loop peptide, raising the HDX rate for this peptide to that seen in wtPTEN.

### Membrane interaction involves exposed loops on both the phosphatase and the C2 domain

To understand the molecular details of how active PTEN interacts with membranes, we carried out HDX–MS experiments on the active wtPTEN in the presence and absence of lipid vesicles. Upon interaction with lipid vesicles, large reductions in the HDX rate were observed around the N-terminus of PTEN (Figure 2C), with numerous peptides showing greater than 25% reduction in HDX. A peptide encompassing residues 2–7 within the PBM had a 42% reduction. Peptide 35–45, which spans the ‘arginine loop’, saw a 39% reduction in HDX, along with peptide 72–81 showing a 19% reduction. Peptide 82–99, in the WPD loop, had a 13% reduction in HDX upon membrane binding. In addition, peptide 155–177, the TI loop, showed a 7% reduction in HDX.

The C2 domain also shows reductions in HDX for two C2 loops (CBRs), peptide 201–215 in the CBR1 (8% reduction) and peptide 258–273 in the CBR3 (11% reduction). Finally, peptide 316–330, incorporating the Cα2 loop, shows an 11% reduction in HDX when PTEN is bound to membrane. Our results using HDX–MS on non-labelled intact PTEN indicate that both the phosphatase and the C2 domain play roles in binding and orienting PTEN on membranes.

### HDX–MS of PTEN-L indicates an ordered segment in the intrinsically disordered N-terminal extension

**Figure 3:**
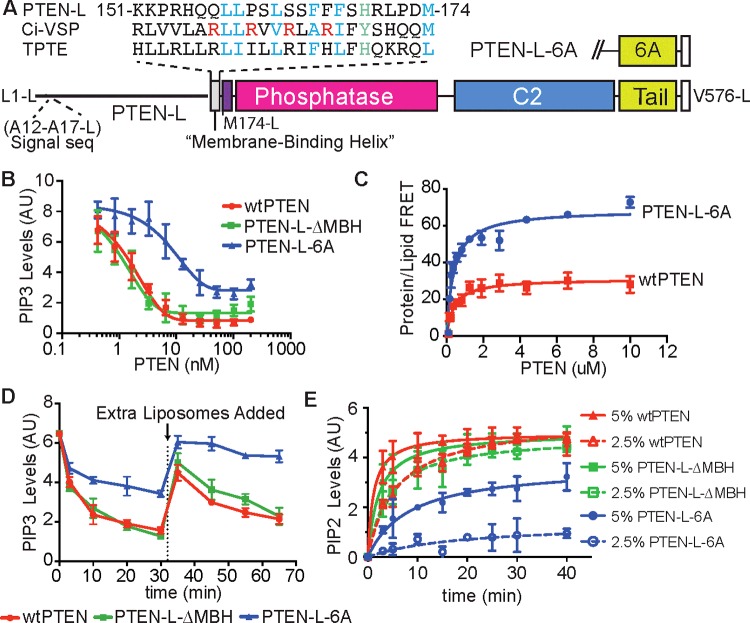
Characterization of PTEN-L (**A**) Schematic representation of PTEN-L. The 173-residue N-terminal extension contains the six alanine residue signal sequence. The predicted α-helix between residues 151-L and 174-L (highlighted) has sequence similarity with the PTEN-family members Ci-VSP and TPTE (transmembrane phosphatase with tensin homology), identified here as the MBH. (**B**) Phosphatase activity against lipid vesicles containing a long-chain PIP_3_ substrate. PIP_2_-containing vesicles (5%) were incubated with PI3K to generate PIP_3_, which was subsequently dephosphorylated with various concentrations of PTEN. Experiments shown in (**B**)–(**E**) were conducted at least three times in triplicate. (**C**) Protein/lipid FRET of PTEN-L-6A and wtPTEN. (**D**) Interfacial kinetics experiment, showing different behaviours of wtPTEN, PTEN-L and PTEN-L-ΔMBH. The reactions were conducted with 10 nM protein for 30 min. At this point the reactions were supplemented with more PIP_3_-containing liposomes to a 4-fold molar excess of liposomes to protein. (**E**) Surface dilution of PIP_3_ also highlights the different interfacial kinetics of wtPTEN, PTEN-L and PTEN-L-ΔMBH. The bulk concentration of PIP_3_ was kept constant by varying the concentration of the carrier lipids. The surface concentration of PIP_3_ was altered between 5% or 2.5%. Although wtPTEN and PTEN-L-ΔMBH show only minor perturbations in phosphatase rate between the two surface concentrations of PIP_3_, PTEN-L sees a drastic reduction in phosphatase activity upon dilution of PIP_3_’s surface concentration.

PTEN-L-6A was purified from Sf9 cells, as attempts to dephosphorylate PTEN-L were unsuccessful. HDX–MS analysis of PTEN-L-6A (Supplementary Figure S9A*)* indicated that the first 145 residues of PTEN-L were intrinsically disordered as previously predicted [31], exhibiting very high levels of HDX–MS (>50% exchange at 3 s of exchange at 0°C). However, one region of the N-terminal extension of PTEN-L, residues 145–176, was below this threshold, suggesting that this region may be folded and forming an α-helix, as suggested by secondary structure prediction programs. Interestingly, this region has sequence similarity to the ‘S4’ transmembrane helix of Ci-VSP (*Ciona intestinalis* Voltage Sensitive Phosphatase), a paralogue of PTEN, further indicating that this region might form an α-helix (Figure 3A). We refer to this region as the ‘membrane-binding helix (MBH)’ for reasons detailed below.

The PBM and the ‘arginine loop’ of PTEN-L-6A showed decreases in HDX compared with wtPTEN (Supplementary Figure S9A). The PBM (residues 177–194 in PTEN-L, 4–21 in PTEN) had a 15% decrease, whereas the arginine loop, (residues 208–217 in PTEN-L, 35–44 in PTEN) had a 7% decrease, suggesting a change in conformation in these regions. Additionally, the C2/PTP interdomain region of PTEN-L exhibited increases in HDX when compared with wtPTEN, suggesting a breakdown of the contacts in this region. A variant of PTEN-L with a deleted MBH (residues Ala^141^-L to Met^174^-L deleted), PTEN-L-ΔMBH, had a HDX profile almost identical with that of wtPTEN (Supplementary Figures S9C and S9D).

### The MBH of PTEN-L causes PTEN-L to become a membrane scooter rather than a hopper

PTEN-L-6A was as active as dephosphorylated wtPTEN against soluble diC_8_-PIP_3_ (Supplementary Figure S9B), but not as active against liposome-incorporated PIP_3_ (Figure 3B). Deleting the MBH (PTEN-L-ΔMBH) increased activity against membrane incorporated PIP_3_ to a level similar to wtPTEN. Despite its apparent lower activity in hydrolysing membrane PIP_3_, protein/lipid FRET measurements indicated that PTEN-L-6A bound to liposomes more tightly than wtPTEN (Figure 3C). We postulated that the reduced activity might be because PTEN-L less readily dissociates from vesicles and cannot move to new PIP_3_-containing vesicles, i.e., PTEN-L works in a scooting rather than hopping mode of catalysis [43]. To determine the interfacial kinetic mode of PTEN-L, we conducted two modified PIP_3_ liposome assays. In the first assay, a second aliquot of substrate was added to produce a 4-fold excess of liposomes relative to PTEN after the initial reaction had completed (Figure 3D). Surprisingly, PTEN-L and wtPTEN exhibited different interfacial kinetic behaviour. The addition of fresh substrate caused a burst of activity with wtPTEN, whereas PTEN-L failed to dephosphorylate the additional liposomes, indicating that wtPTEN works in a ‘hopping’ mode of interfacial kinetics, in agreement with previous studies [44], whereas PTEN-L-6A works in a ‘scooting’ mode. In a second assay, we measured the phosphatase rate of wtPTEN and PTEN-L with the PIP_3_ surface concentration of the membrane at 5% and 2.5%, while maintaining a constant bulk PIP_3_ concentration. The reduction in surface concentration greatly affected the phosphatase rate of PTEN-L (Figure 3E), whereas wtPTEN was affected to a much smaller extent. The results indicate that PTEN-L is carrying out a scooting catalysis, whereas wtPTEN shows predominately hopping behaviour. In both experiments, PTEN-L-ΔMBH exhibited the same behaviour as wtPTEN, suggesting that the MBH is responsible for this change in interfacial kinetics.

### PTEN-L has a vastly altered membrane-binding footprint

**Figure 4:**
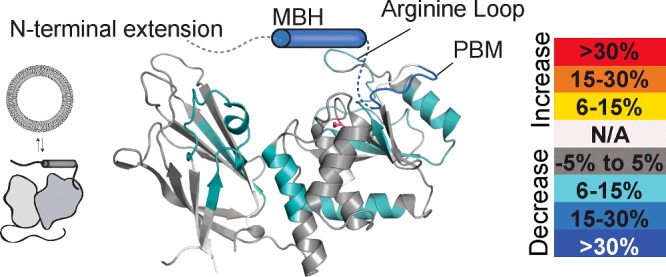
Changes in HDX observed on PTEN-L-6A in the presence of PIP_2_-containing vesicles The N-terminal extension is shown as a cartoon, with the MBH as a cylinder. Areas with no peptide coverage are shown as white (N/A).

To determine how the N-terminal extension of PTEN-L caused a change in interfacial kinetics, we compared HDX of PTEN-L-6A in the presence and absence of lipid vesicles (Figure 4; Supplementary Figure S8). The membrane-binding footprint was very different from that of wtPTEN, with the MBH having the largest changes in HDX (MBH peptides 150–162, 166–176 and 167–176 decreasing 12%, 20% and 22% respectively, PTEN-L numbering). Additionally, PTEN-L showed decreases in HDX in areas associated with membrane binding for wtPTEN, although most changes were of a smaller magnitude. For example, a PBM-containing peptide (residues 177–194 in PTEN-L, 4–21 in PTEN) decreased 12% in PTEN-L as compared with 28% for the same peptide in PTEN, a peptide covering the arginine loop (208–215 in PTEN-L, 35–42 in PTEN) decreased 11% when PTEN-L binds to liposomes, compared with 32% in PTEN and a peptide spanning the Cα2 loop (6% decrease for 492–514 in PTEN-L, 11% decrease for 319–341 in PTEN). Additionally, the CBR3 loop of PTEN-L no longer showed a decrease in exchange when bound to membranes.

## DISCUSSION

PTEN plays important tumour-suppressing roles as both a lipid and a protein phosphatase [8], and the membrane permeability of PTEN-L presents an opportunity to commandeer these activities as a therapeutic agent. We have shown that phosphorylation of the C-terminal tail switches PTEN's substrate specificity and that a helix within the N-terminal extension of PTEN-L markedly alters the enzyme kinetics on membranes. Development of PTEN-L as a therapeutic agent will require consideration of these two disordered segments and the effect they have on ability of PTEN to function as a tumour suppressor.

We find that fully phosphorylated PTEN is completely inactive and incapable of binding membranes. However, selective dephosphorylation of only two residues, Thr^366^ and Ser^370^, causes the active site of PTEN to become partially exposed, allowing cytosolic PTEN to dephosphorylate its growing list of soluble substrates (both phosphoserine/threonine and phosphotyrosine residues) [4,45,46], including CREB [47], IRS1 [8], Shc [48], components of the MAPK pathway [49] and Dishevelled [50]. Furthermore, this selective activity may have an impact on an emerging role of PTEN in dephosphorylating soluble protein-bound PIP_3_, such as PIP_3_ bound to SF-1 within the nucleus [51]. Our data indicate that phosphorylation of Thr^366^ and Ser^370^ alone is not sufficient to occlude the active site, as Phos-PTEN-4A was still capable of dephosphorylating soluble substrates. HDX–MS suggests that dephosphorylation of Thr^366^ and Ser^370^ results in localized conformational changes; however, these localized changes are apparently not great enough to have been detected by using access to alkaline phosphatase as a measure of conformational change [35].

By using HDX–MS to map both the membrane-binding interface and the interface between the phosphorylated C-terminal tail with the rest of PTEN, we determined the structural basis for the substrate switch of PTEN. The phosphorylated tail interacts predominantly with the N-terminal PBM, the active site of the phosphatase domain and the Cα2 loop of the C2 domain. Previous studies have determined the CBR3 loop of the C2 domain as the binding partner for the phosphorylated tail, through either electrostatic [35,52] or non-electrostatic cryptic binding motifs [14,37]. However, these studies are based on the mutagenesis or deletion of the CBR3 loop. Our HDX–MS experiments, carried out on the native protein, are not consistent with the phosphorylated tail interacting with CBR3. This is in agreement with a previous study that highlighted the need for an intact active site and PBM to allow for the intramolecular binding event [22]. Here, HDX shows that the mechanism for inhibition involves occlusion of the active site by the phosphorylated tail, preventing substrate catalysis, rather than the prevention of PTEN membrane binding through occlusion of the CBR3 loop. The proximity of pThr^366^ and pSer^370^ to the active site may explain their propensity for auto-dephosphorylation [38].

Our results also provide a comprehensive picture of how PTEN binds to membranes. For membrane binding and PTEN activity assays, we used a complex mixture of lipids that mimics the composition of the plasma membrane [41], as studies have shown that altering the relative proportions of various lipid moieties can effect both PTEN's ability to interact with the membrane and its subsequent lipid phosphatase activity [35,44]. Attention has focused on the CBR3 loop as the dictating element of membrane interaction [9,10,15,35,37,52], but we observe a more complex picture, with very large reductions in HDX throughout the phosphatase domain and similar reductions in HDX in two of the three CBR loops, as well as in the Cα2 loop. Mutations in the Cα2 loop decrease activity *in vitro* and membrane localization in cells [13,53]. Within the phosphatase domain, our data highlight the membrane-binding contribution of the ‘arginine loop’, which forms a positively charged patch, composed of Arg^41^, Arg^47^, Arg^74^ and Lys^80^. Finally, although both the WPD and the TI loops are protected by membrane binding, the P loop, which contains the catalytically crucial cysteine, is not. These disparate regions of PTEN, i.e. the arginine loop, the TI loop, the CBR3 loop, the Cα2 loop and the PBM, have all been implicated in membrane interaction as modelled by molecular dynamic simulations [54]. Together, our results indicate that a large surface of PTEN interacts with the membrane.

From our results, we propose that PTEN can exist in three distinct activation states (Figure 2D) depending on the phosphorylation status of the tail: a fully phosphorylated state that is inactive against both lipid and protein substrates, a partially dephosphorylated state (lacking phosphates on Ser^366^ and Thr^370^), active only against soluble substrates including phosphoproteins, and a fully dephosphorylated state that is active against both soluble and liposome-incorporated substrates. The activation states are mediated by intramolecular inhibitory interactions between the tail and the C2 and phosphatase domains that occlude both the membrane-binding region as well as the active site.

Whether these three activation states are applicable to PTEN-L is not yet clear. PTEN-L is an N-terminally extended translational variant of PTEN, which was shown to be capable of exiting the cell and entering a neighbouring cell, down-regulating their p-Akt levels [29] and possibly localizing to mitochondria to regulate cytochrome *c* oxidase activity [30]. We have found that the N-terminal elongation of PTEN-L is not entirely devoid of secondary structure and that an ordered region, most likely an α-helix, exists between residues 151-L and 174-L. This region resembles the transmembrane S4-helix of voltage-sensitive phosphatase Ci-VSP, a PTEN family member. Upon membrane depolarization, the S4-helix changes conformation, causing the rest of the enzyme (which shares strong similarity with PTEN) to adopt an active conformation on the membrane [55]. The exact mechanism of how this conformational change is communicated to the rest of the enzyme is not well understood, although part of the linker, corresponding in sequence to the PBM (residues Arg^13^/Lys^14^ in PTEN), may interact with the TI loop of the phosphatase domain, thereby acting as a gate for activity [56].

**Figure 5:**
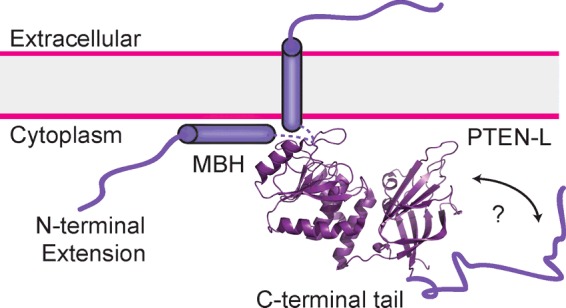
Possible membrane-binding mode of PTEN-L The MBH of PTEN-L may interact with the membrane surface or may be a transmembrane helix. This interaction changes the interfacial catalytic mechanism from a ‘hopping’ mode, characteristic of PTEN to a ‘scooting’ mode, characteristic of PTEN-L.

Using HDX–MS, we have shown that this 151-L–174-L helix (MBH) in PTEN-L is strongly protected by liposomes, suggesting an interaction with the membrane. The MBH alters both the interfacial kinetics of the enzyme and the protein/membrane interface (Figures 3B–3E and 4). The presence of the MBH causes PTEN-L to function on membranes in a ‘scooting’ mode rather than a ‘hopping’ mode that is characteristic of PTEN, i.e. PTEN-L seems to remain attached to the membrane, diffusing two-dimensionally, rather than readily diffusing between separate membranes like PTEN. Deletion of the MBH caused PTEN-L to revert to the same hopping behaviour as PTEN. PTEN-L's scooting mechanism may have important cellular implications, such as causing an enrichment of PTEN-L on certain membranes. Whether the increase in affinity of PTEN-L for the membrane imparted by MBH is sufficient to counter the decrease in membrane affinity observed on phosphorylation of the C-terminus of PTEN is not known. Whether the MBH is a transmembrane helix or lies against the membrane surface, as recently proposed for a helix found in the PBM [57], has yet to be determined (Figure 5). Our data also show that the PBM is structurally altered and stabilized in PTEN-L compared with PTEN, even in the absence of membranes, suggesting that there may be a network of interactions that link the MBH to the PBM and reduces the role of the PBM in the membrane binding of PTEN-L. Furthermore, the CBR3 and CBR1 loops of the PTEN-L C2 domain do not engage membranes, suggesting that the catalytic domain may be sitting on the membrane differently than PTEN.

PTEN-L and PTEN exhibit complex mechanisms of interdependent changes in conformation, membrane binding and substrate specificity that are mediated by two intrinsically disordered tails. A combination of differential phosphorylations of the C-terminal tail and extension of the N-terminus provides a large repertoire of active states with different membrane-binding properties. The use of recombinant PTEN-L has been proposed as a potentially novel therapeutic strategy to introduce PTEN activity in PTEN-deficient cancers and the present study underscores the complexity of regulation of PTEN and PTEN-L. Further work on the mechanistic aspects that underpin PTEN and PTEN-L function is essential to enable their use as a therapeutic agent.

## Abbreviations

CBR: calcium-binding region
Ci-VSP: *Ciona intestinalis* Voltage Sensitive Phosphatase
GSK3β: glycogen synthase kinase 3β
HDX: hydrogen/deuterium exchange
IP_4_: inositol tetrakisphosphate
IRS1: insulin receptor substrate 1
LC-MS: liquid chromatography-mass spectrometry
MBH: membrane-binding helix
PBM: PIP_2_-binding motif
Phospho-wtPTEN: phosphorylated wild-type PTEN
PI3K: phosphoinositide 3-kinase
PIP_2_: phosphatidylinositol 4,5-bisphosphate
PIP_3_: phosphatidylinositol 3,4,5-trisphosphate
Phos-PTEN-2A/4A: phosphorylated PTEN-2A/4A mutant
PS: phosphatidylserine
PTEN-ΔTail: truncated PTEN lacking the tail
PTP: protein tyrosine phosphatase
PTEN: phosphatase and tensin homologue deleted on chromosome 10
PTEN-L: PTEN-long
SEC–MALS: size-exclusion chromatography–multi-angle light scattering
TBST: TBS/Tween 20 solution
TCEP: tris-(2-carboxyethyl)-phosphine
TEV: tobacco etch virus
TPTE: transmembrane phosphatase with tensin homology
UPLC: ultra-performance liquid chromatography
wtPTEN: dephosphorylated wild-type PTEN

## References

[1] Liaw D., Marsh D.J., Li J., Dahia P.L.M., Wang S.I., Zheng Z., Bose S., Call K.M., Tsou H.C., Peacoke M. (1997). Germline mutations of the PTEN gene in Cowden disease, an inherited breast and thyroid cancer syndrome. Nat. Genet..

[2] Steck P.A., Pershouse M.A., Jasser S.A., Yung W.K.A., Lin H., Ligon A.H., Langford L.A., Baumgard M.L., Hattier T., Davis T. (1997). Identification of a candidate tumour suppressor gene, MMAC1, at chromosome 10q23.3 that is mutated in multiple advanced cancers. Nat. Genet..

[3] Yuan T.L., Cantley L.C. (2008). PI3K pathway alterations in cancer: variations on a theme. Oncogene.

[4] Myers M.P., Stolarov J.P., Eng C., Li J., Wang S.I., Wigler M.H., Parsons R., Tonks N.K. (1997). P-TEN, the tumor suppressor from human chromosome 10q23, is a dual-specificity phosphatase. Proc. Natl. Acad. Sci. U.S.A..

[5] Myers M.P., Pass I., Batty I.H., Van der Kaay J., Stolarov J.P., Hemmings B.A., Wigler M.H., Downes C.P., Tonks N.K. (1998). The lipid phosphatase activity of PTEN is critical for its tumor supressor function. Proc. Natl. Acad. Sci. U.S.A..

[6] Maehama T., Dixon J.E. (1998). The tumor suppressor, PTEN/MMAC1, dephosphorylates the Lipid second messenger, phosphatidylinositol 3,4,5-trisphosphate. J. Biol. Chem..

[7] Cantley L.C., Neel B.G. (1999). New insights into tumor suppression: PTEN suppresses tumor formation by restraining the phosphoinositide 3-kinase/AKT pathway. Proc. Natl. Acad. Sci. U.S.A..

[8] Shi Y., Wang J., Chandarlapaty S., Cross J., Thompson C., Rosen N., Jiang X. (2014). PTEN is a protein tyrosine phosphatase for IRS1. Nat. Struct. Mol. Biol..

[9] Suzuki A., la Pompa, de J.L., Stambolic V., Elia A.J., Sasaki T., Barrantes I.D.B., Ho A., Wakeham A., ltie A., Khoo W. (1998). High cancer susceptibility and embryonic lethality associated with mutation of the PTEN tumor suppressor gene in mice. Curr. Biol..

[10] Salmena L., Carracedo A., Pandolfi P.P. (2008). Tenets of PTEN tumor suppression. Cell.

[11] Berger A.H., Knudson A.G., Pandolfi P.P. (2011). A continuum model for tumour suppression. Nature.

[12] Papa A., Wan L., Bonora M., Salmena L., Song M.S., Hobbs R.M., Lunardi A., Webster K., Ng C., Newton R.H. (2014). Cancer-associated PTEN mutants act in a dominant-negative manner to suppress PTEN protein function. Cell.

[13] Lee J.-O., Yang H., Georgescu M.-M., Di Cristofano A., Maehama T., Shi Y., Dixon J.E., Pandolfi P., Pavletich N.P. (1999). Crystal structure of the PTEN tumor suppressor. Cell.

[14] Walker S.M., Leslie N.R., Perera N.M., BATTY I.H., Downes C.P. (2004). The tumour-suppressor function of PTEN requires an N-terminal lipid-binding motif. Biochem. J..

[15] Yang J., Niu T., Zhang A., Mishra A.K., Zhao Z.J., Zhou G.W. (2001). Relation between the flexibility of the WPD loop and the activity of the catalytic domain of protein tyrosine phosphatase SHP-1. J. Cell. Biochem..

[16] Rodriguez-Escudero I., Oliver M.D., Andres-Pons A., Molina M., Cid V.J., Pulido R. (2011). A comprehensive functional analysis of PTEN mutations: implications in tumor- and autism-related syndromes. Hum. Mol. Genet..

[17] Brandão T.A.S., Johnson S.J., Hengge A.C. (2012). The molecular details of WPD-loop movement differ in the protein-tyrosine phosphatases YopH and PTP1B. Arch. Biochem. Biophys..

[18] Xiao Y., Yeong Chit Chia J., Gajewski J.E., Sio Seng Lio D., Mulhern T.D., Zhu H.-J., Nandurkar H., Cheng H.-C. (2007). PTEN catalysis of phospholipid dephosphorylation reaction follows a two-step mechanism in which the conserved aspartate-92 does not function as the general acid — mechanistic analysis of a familial Cowden disease-associated PTEN mutation. Cell. Signal..

[19] Lu J., Jeong H., Kong N., Yang Y., Carroll J., Luo H.R., Silberstein L.E., YupoMa, Chai L. (2009). Stem cell factor SALL4 represses the transcriptions of PTEN and SALL1 through an epigenetic repressor complex. PLoS One.

[20] Song M.S., Salmena L., Pandolfi P.P. (2012). The functions and regulation of the PTEN tumour suppressor. Nat. Rev. Mol. Cell. Bio..

[21] Vazquez F., Grossman S.R., Takahashi Y., Rokas M.V., Nakamura N., Sellers W.R. (2001). Phosphorylation of the PTEN tail acts as an inhibitory switch by preventing its recruitment into a protein complex. J. Biol. Chem..

[22] Rahdar M., Inoue T., Meyer T., Zhang J., Vazquez F., Devreotes P.N. (2009). A phosphorylation-dependent intramolecular interaction regulates the membrane association and activity of the tumor suppressor PTEN. Proc. Natl. Acad. Sci. U.S.A..

[23] Okumura K. (2006). PCAF modulates PTEN activity. J. Biol. Chem..

[24] Lee S.R., Yang K.S., Kwon J., Lee C., Jeong W., Rhee S.G. (2002). Reversible inactivation of the tumor suppressor PTEN by H_2_O_2_. J. Biol. Chem..

[25] Wang X., Trotman L.C., Koppie T., Alimonti A., Chen Z., Gao Z., Wang J., Erdjument-Bromage H., Tempst P., Cordon-Cardo C. (2007). NEDD4-1 is a proto-oncogenic ubiquitin ligase for PTEN. Cell.

[26] Huang J., Yan J., Zhang J., Zhu S., Wang Y., Shi T., Zhu C., Chen C., Liu X., Cheng J. (2012). SUMO1 modification of PTEN regulates tumorigenesis by controlling its association with the plasma membrane. Nat. Commun..

[27] Putz U., Howitt J., Doan A., Goh C.P., Low L.H., Silke J., Tan S.S. (2012). The tumor suppressor PTEN is exported in exosomes and has phosphatase activity in recipient cells. Sci. Signal..

[28] Pulido R., Baker S.J., Barata J.T., Carracedo A., Cid V.J., Chin-Sang I.D., Dave V., Hertog, den J., Devreotes P., Eickholt B.J. (2014). A unified nomenclature and amino acid numbering for human PTEN. Sci. Signal..

[29] Hopkins B.D., Fine B., Steinbach N., Dendy M., Rapp Z., Shaw J., Pappas K., Yu J.S., Hodakoski C., Mense S. (2013). A secreted PTEN phosphatase that enters cells to alter signaling and survival. Science.

[30] Liang H., He S., Yang J., Jia X., Wang P., Chen X., Zhang Z., Zou X., McNutt M.A., Shen W.H. (2014). PTENα, a PTEN isoform translated through alternative initiation, regulates mitochondrial function and energy metabolism. Cell Metab..

[31] Malaney P., Uversky V.N., Davé V. (2013). The PTEN Long N-tail is intrinsically disordered: increased viability for PTEN therapy. Mol. BioSyst..

[32] Johnston S.B., Raines R.T. (2015). Catalysis by the tumor-suppressor enzymes PTEN and PTEN-L. PLoS One.

[33] Al-Khouri A.M., Ma Y., Togo S.H., Williams S., Mustelin T. (2005). Cooperative phosphorylation of the tumor suppressor phosphatase and tensin homologue (PTEN) by casein kinases and glycogen synthase kinase 3β. J. Biol. Chem..

[34] Cordier F., Chaffotte A., Terrien E., Préhaud C., Theillet F.-X., Delepierre M., Lafon M., Buc H., Wolff N. (2012). Ordered phosphorylation events in two independent cascades of the PTEN C-tail revealed by NMR. J. Am. Chem. Soc..

[35] Bolduc D., Rahdar M., Tu-Sekine B., Sivakumaren S.C., Raben D., Amzel L.M., Devreotes P., Gabelli S.B., Cole P. (2013). Phosphorylation-mediated PTEN conformational closure and deactivation revealed with protein semisynthesis. Elife.

[36] Torres J., Pulido R. (2001). The tumor suppressor PTEN is phosphorylated by the protein kinase CK2 at Its C terminus: implications for PTEN stability to proteasome-mediated degradation. J. Biol. Chem..

[37] Odriozola L., Singh G., Hoang T., Chan A.M. (2007). Regulation of PTEN activity by its carboxyl-terminal autoinhibitory domain. J. Biol. Chem..

[38] Tibarewal P., Zilidis G., Spinelli L., Schurch N., Maccario H., Gray A., Perera N.M., Davidson L., Barton G.J., Leslie N.R. (2012). PTEN protein phosphatase activity correlates with control of gene expression and invasion, a tumor-suppressing phenotype, but not with AKT activity. Sci. Signal..

[39] Maccario H., Perera N.M., Davidson L., Downes C.P., Leslie N.R. (2007). PTEN is destabilized by phosphorylation on Thr 366. Biochem. J..

[40] Nalefski E.A., Slazas M.M., Falke J.J. (1997). Ca 2+-signaling cycle of a membrane-docking C2 domain †. Biochemistry.

[41] Vance J., Steenbergen R. (2005). Metabolism and functions of phosphatidylserine. Prog. Lipid Res..

[42] Burke J.E., Perisic O., Masson G.R., Vadas O., Williams R.L. (2012). Oncogenic mutations mimic and enhance dynamic events in the natural activation of phosphoinositide 3-kinase p110α (PIK3CA). Proc. Natl. Acad. Sci. U.S.A..

[43] Jain M.K., Berg O.G. (1989). The kinetics of interfacial catalysis by phospholipase A2 and regulation of interfacial activation: hopping versus scooting. Biochim Biophys Acta.

[44] McConnachie G., Pass I., Walker S.M., Downes C.P. (2003). Interfacial kinetic analysis of the tumour suppressor phosphatase, PTEN: evidence for activation by anionic phospholipids. Biochem. J..

[45] Vanhaesebroeck B. (1999). Autophosphorylation of p110delta phosphoinositide 3-kinase: a new paradigm for the regulation of lipid kinases *in vitro* and *in vivo*. EMBO J..

[46] Dey N., Crosswell H.E., De P., Parsons R., Peng Q., Su J.D., Durden D.L. (2008). The protein phosphatase activity of PTEN regulates Src family kinases and controls glioma migration. Cancer Res..

[47] Gu T., Zhang Z., Wang J., Guo J., Shen W.H., Yin Y. (2011). CREB is a novel nuclear target of PTEN phosphatase. Cancer Res..

[48] Schneider E., Keppler R., Prawitt D., Steinwender C., Roos F.C., Thüroff J.W., Lausch E., Brenner W. (2011). Migration of renal tumor cells depends on dephosphorylation of Shc by PTEN. Int. J. Oncol..

[49] Nakdimon I., Walser M., Fröhli E., Hajnal A. (2012). PTEN Negatively Regulates MAPK Signaling during *Caenorhabditis elegans* vulval development. PLoS Genet..

[50] Shnitsar I., Bashkurov M., Masson G.R., Ogunjimi A.A., Mosessian S., Cabeza E.A., Hirsch C.L., Trcka D., Gish G., Jiao J. (2015). PTEN regulates cilia through Dishevelled. Nat. Commun..

[51] Blind R.D., Suzawa M., Ingraham H.A. (2012). Direct modification and activation of a nuclear receptor-PIP2 complex by the inositol lipid kinase IPMK. Sci. Signal..

[52] Nguyen H.N., Afkari Y., Senoo H., Sesaki H., Devreotes P.N., Iijima M. (2013). Mechanism of human PTEN localization revealed by heterologous expression in Dictyostelium. Oncogene.

[53] Yasui M., Matsuoka S., Ueda M. (2014). PTEN hopping on the cell membrane is regulated via a positively-charged C2 domain. PLoS Comput. Biol..

[54] Lumb C.N., Sansom M.S. P. (2013). Defining the membrane-associated state of the PTEN tumor suppressor protein. Biophys. J..

[55] Li Q., Wanderling S., Paduch M., Medovoy D., Singharoy A., McGreevy R., Villalba-Galea C.A., Hulse R.E., Roux B., Schulten K. (2014). Structural mechanism of voltage-dependent gating in an isolated voltage-sensing domain. Nat. Struct. Mol. Biol..

[56] Hobiger K., Utesch T., Mroginski M.A., Seebohm G., Friedrich T. (2013). The linker pivot in Ci-VSP: the key to unlock catalysis. PLoS One.

[57] Wei Y., Stec B., Redfield A.G., Weerapana E., Roberts M.F. (2015). Phospholipid-binding sites of phosphatase and tensin homolog (PTEN). J. Biol. Chem..

[58] Dowler S., Kular G., Alessi D.R. (2002). Protein lipid overlay assay. Sci. Signal..

